# Assessment of left ventricular energy loss using vector flow mapping in patients with stages 1–3 chronic kidney disease

**DOI:** 10.1186/s12872-020-01640-9

**Published:** 2020-08-02

**Authors:** Xiaoxue Chen, Yueheng Wang, Wei Wang, Lijun Yuan, Zhengqin Qi, Degang Song

**Affiliations:** 1 Department of Cardiac Ultrasound, Second Hospital of Hebei Medical University, 215 Hepingxi Road, Shijiazhuang, 050000 Hebei China; 2 First Hospital of Qinhuangdao, Qinhuangdao, 066000 Hebei China

**Keywords:** Intracardiac hemodynamics, Vector flow mapping, Energy loss, Stage 1–3 chronic kidney disease, Hypertension

## Abstract

**Background:**

Patients with chronic kidney disease (CKD) experience abnormality of intracardiac blood flow status during early-stages of disease. Left ventricular energy loss (EL) derived from vector flow mapping (VFM) represents fluid energy lost as heat in left ventricle and had been used to detect intracardiac blood flow efficiency. We aimed to evaluate the left ventricular EL in stage 1–3 CKD patients, and explored whether hypertension, a main cardiovascular risk, deteriorate the abnormality of intracardiac blood flow status.

**Methods:**

Transthoracic echocardiography was performed in 41 controls and 48 patients with stages 1–3 CKD. CKD patients consisted a subgroup with no hypertension, a subgroup with well-controlled hypertension and a subgroup with poorly controlled hypertension. The EL were calculated in the left ventricle using VFM analysis from the apical 3-chamber view. Furthermore, the correlation and stepwise multiple regression analysis were used to explore the potential independent predictors of left ventricular EL.

**Results:**

Compared with controls, stage 1–3 CKD patients showed increased left ventricular EL during total diastole, late diastole, total systole, isovolumic contraction and ejection. CKD patients with poorly controlled hypertension had higher left ventricular EL compared to the other CKD subgroups. Additionally, the ratio of mitral early filling wave peak velocity and early mitral annular peak velocity on septal side, mitral early filling wave peak velocity, and left ventricular mass index were independent predictors of the diastolic EL; whereas systolic blood pressure and left ventricular mass index were independent predictors of the systolic EL.

**Conclusions:**

Left ventricular EL was a useful echocardiographic parameter to evaluate the impaired intracardiac blood flow efficiency in patients with stages 1–3 CKD. Hypertension was a crucial contributor for intracardiac blood flow abnormality. This study might provide valuable clinical data to discern cardiac dysfunction and reduce the cardiovascular risk in early-stage CKD.

## Background

Chronic kidney disease (CKD) affects about one in ten adults [1], and is principally caused by diabetes, or glomerulonephritis in china [2]. Patients with stages 1–3 CKD have a high cardiovascular morbidity and mortality compared with healthy people [3, 4]. However, the changes in cardiac structure and volume are relatively slight in stage 1–3 CKD patients who have a preserved cardiac ejection fraction [5, 6]. Actually, diffuse myocardial interstitial fibrosis indeed occurs in mild to moderate CKD [7], resulting in abnormality of intracardiac blood flow status [8]. Therefore, accurate and reproducible evaluation on the early cardiac dysfunction were urgently need from the perspective of fluid dynamics in stages 1–3 CKD when the best opportunity of effective treatment exists.

Cardiac magnetic resonance, contrast particle imaging velocimetry and vector flow mapping (VFM) are mainly techniques for evaluating the intracardiac blood flow status. Compared to the other two techniques, VFM is more suitable for clinical application owing to its convenient, non-invasive and inexpensive characteristics [9, 10], and its accuracy has been confirmed by particle imaging velocimetry [11, 12]. Additionally, VFM, as a new echocardiographic technique, has no angle dependence or limitation of the region of interest, making it superior to traditional Doppler technology [13]. Energy loss (EL) is an important hemodynamic parameter based on VFM technology, representing the amount of fluid energy that was lost as heat in the heart and indicating the efficiency of intracardiac blood flow [14]. If the intracardiac blood flow displays pathological pattern, such as turbulence, jet or adverse direction, its EL increases correspondingly [15]. Monitoring EL by VFM enables quantitative evaluation on the change of intraventricular hemodynamics. Left ventricular EL has been considered as a novel clinical index to detect cardiac dysfunction in diabetic [15, 16] and a useful tool for the detection of subclinical cardiac dysfunction in patients with hypertrophic cardiomyopathy [17]. Growing evidences show EL evaluation has received increasing attention in clinical practice [18, 19], it might provide a new perspective on heart research.

Left ventricular EL bas been shown its efficacy in evaluation of intracardiac fluid dynamics in uremic hearts [20]. Although patients with stages 1–3 CKD seems not to experience volume shifts and obvious left ventricular hypertrophy compared with end-stage CKD patients, intracardiac blood flow status has pathologically changed in their heart partly owing to the diffuse myocardial interstitial fibrosis. We speculated patients with stages 1–3 CKD experience impaired intracardiac blood flow efficiency, and left ventricular EL could be a novel echocardiographic parameter for assessing their cardiac dysfunction in terms of fluid mechanics, but the related research has not yet been known. Importantly, hypertension, as a main cardiovascular risk factor, is frequently detected in patients with mild to moderate CKD, unfortunately, it does not get enough attention in real-world until a serious cardiovascular event unexpectedly occurs [21, 22]. Additionally, whether the hypertension have further effect on the blood flow efficiency in patients with stages 1–3 CKD remains largely unknown. Therefore, we quantitatively detected the left ventricular EL in stage 1–3 CKD patients using VFM, and further focused on the change of left ventricular EL in patients with poorly controlled hypertension. This study was expected to provide valuable clinical data to reduce the disproportionately risk of cardiovascular disease and slow, or halt the progression of CKD during the early stages.

## Methods

### Study design

Patients with CKD (KDOQI stages 1–3) who accepted professional treatment according to CKD clinical guidelines in the Second Hospital of Hebei Medical University from 2016 to 2019 were invited to participate this study. The criteria for enrollment were stable stage 1–3 CKD patients with primary glomerulonephritis (diagnosis with kidney puncture), adequate echocardiograms with sinus rhythm. Exclusion criteria were clinical evidence of heart failure, substantial valvular stenosis or regurgitation, and a history of angina pectoris or myocardial infarction, congenital heart disease, hypertrophic cardiomyopathy, pulmonary heart disease, essential hypertension and diabetes. Overall, this cross-sectional study recruited 48 CKD patients (KDOQI stages 1–3). To explore whether the hypertension have further effect on the blood flow efficiency in CKD stages 1–3. The patients were divided into three subgroups according to the Global Outcomes (KDIGO) clinical practice guideline for the management of blood pressure in non-dialysis-dependent CKD [23]: patients without hypertension (N-HTN, *n* = 10): Without using antihypertensive drugs, blood pressure < 140/90 mmHg (if age > 60, blood pressure < 150/90 mmHg) or blood pressure < 130/80 mmHg with overt proteinuria; patients with well-controlled hypertension (W-HTN, *n* = 18): Using antihypertensive drugs, blood pressure < 140/90 mmHg (if age > 60, blood pressure < 150/90 mmHg) or blood pressure < 130/80 mmHg with overt proteinuria; patients with poorly controlled hypertension (P-HTN, *n* = 20): Regardless of whether taking antihypertensive drugs, blood pressure ≥ 140/90 mmHg (if age > 60, blood pressure ≥ 150/90 mmHg) or blood pressure ≥ 130/80 mmHg with overt proteinuria. Blood pressure was measured after a rest for at least 10 min in a supine position and averaged from three continuously monitoring on the first 3 days after admission [21]. Forty-one healthy subjects were enrolled as a control group. The clinical data for all participants were obtained before the echocardiographic inspections. Furthermore, designated conventional echocardiographic and clinical parameters were analyzed using correlation and stepwise multiple regression analysis to explore the factors affecting left ventricular EL in stage 1–3 CKD patients. Information on experimental groups was blinded to the researchers who performed echocardiography and data analysis.

### Transthoracic echocardiography

Transthoracic echocardiography was performed by experienced sonographers using a Pro-Sound F75 ultrasound device (Hitachi-Aloka Medical Ltd., Tokyo, Japan) with a UST-52105 probe (1–5 MHz) in all subjects.

The conventional echocardiographic parameters were acquired according to the American Society of Echocardiography guidelines [24]. Left ventricular end-diastolic diameter (LVEDd), left ventricular end-systole diameter (LVEDs), interventricular septal thickness (IVS), and left ventricular posterior wall thickness (LVPW) were measured using M-mode in the parasternal long-axis view. Left ventricular end-diastolic volume (LVEDV), left ventricular end-systolic volume (LVESV), left ventricular ejection fraction (LVEF), and left ventricular mass index (LVMI) were automatically calculated according to predefined formulas. Transmitral Doppler flow was used to measure the peak wave velocities during early filling (E-wave) and late filling (A-wave) in the apical four-chamber view. The peak wave velocity at the mitral valve (E-wave) and the peak myocardial velocities at the mitral annulus on the septal side (e’) during early filling were detected in the apical four-chamber view by dual-mode Doppler imaging, and E/e’ ratio was recorded.

### Image acquisition and principles of vector flow mapping

Color Doppler Images for flow visualization were obtained from an apical three-chamber view in VFM mode, which was available for analyzing offline. The depth, width, angle and spatial-temporal resolution of images were adjusted to obtain the frame rate as high as possible (frame rate > 23 frames/s) while including left ventricle, left ventricular inflow and outflow tract in the color-scan area. Additionally, to mitigate aliasing phenomena, the Nyquist limit about two-dimensional color Doppler imaging should be set high sufficiently. Three cardiac cycles were stored, and the images were analyzed using VFM analysis software (DASRS1; Hitachi Aloka Medical Ltd.) installed in an offline workstation, so as to obtain the velocity vector fields about intraventricular blood flow. In brief, VFM visualizes intraventricular blood flow by velocity vector based on color Doppler imaging and two-dimensional speckle tracking. Color Doppler shows flow velocity in the parallel direction of the echo beam, whereas two-dimensional speckle tracking tracks ventricular wall motion, and blood flow velocity component in the direction perpendicular to the echo beam is calculated by continuity equation, which begins from the vicinity of the cardiac wall [10].

**Fig. 1:**
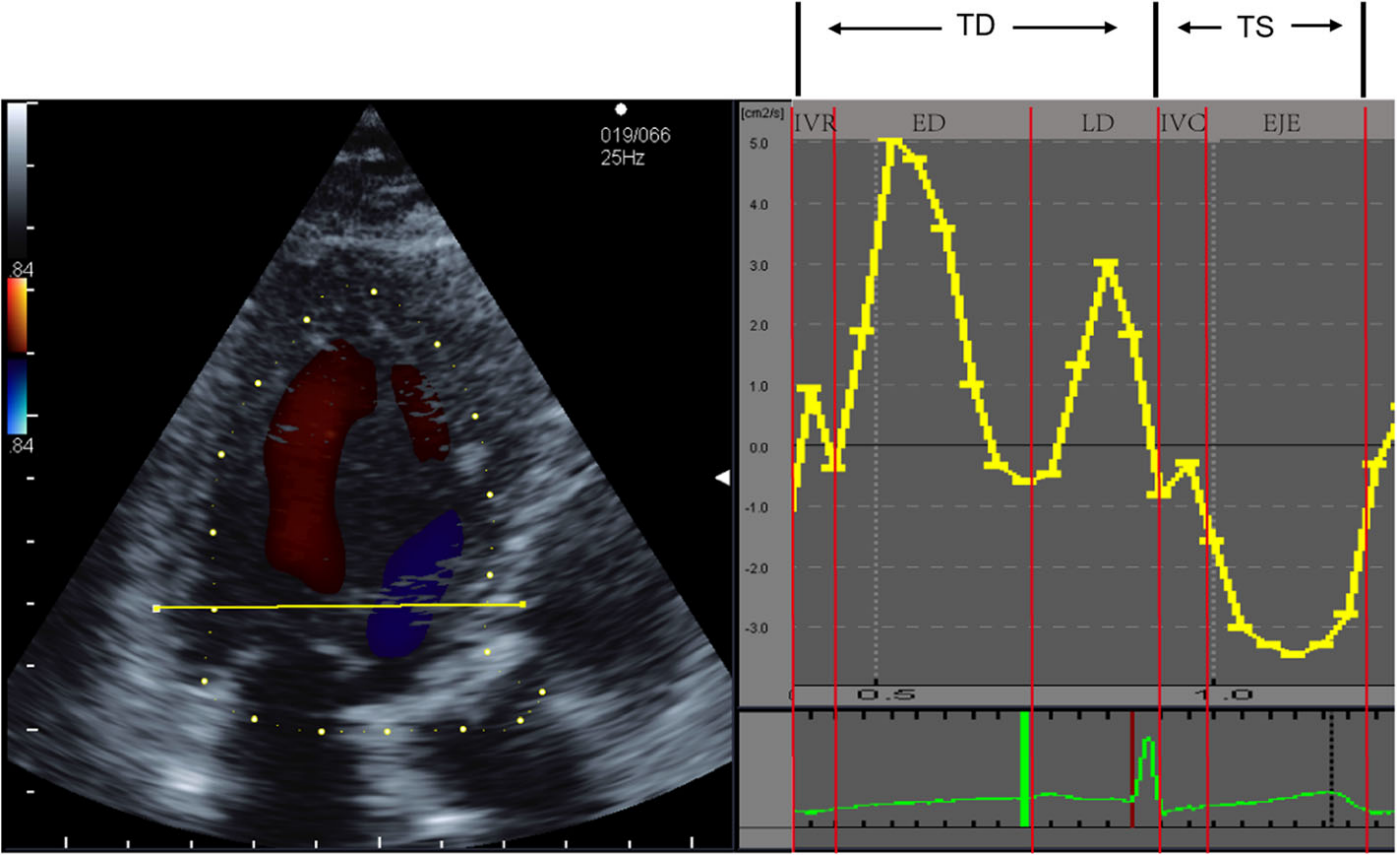
Time-flow curve representing the division (five phases) of a cardiac cycle. IVR starts from the closure of the aortic valve to the opening of the mitral valve, ED starts from the opening of the mitral valve to the opening of the mitral valve once more, LD starts from the opening of the mitral valve once more to its closure, IVC starts from the closure of the mitral valve to the opening of the aortic valve, EJE starts from the opening of the aortic valve to the closure of the aortic valve

The EL was the amount of fluid energy lost and dissipated as heat in the cardiac cavity. From the velocity vector fields of the intracardiac blood flow, EL for every frame of the cine-loop image was calculated by the formula [25] as follow:

$$ \mathrm{EL}={\sum}_{ij}\int \frac{1}{2}\mu \left(\frac{\partial {u}_i}{\partial {x}_j}+\frac{\partial {u}_j}{\partial {x}_i}\right) dv $$

where *μ* is the blood viscosity, u is the component of the velocity vector, i and j are the two-dimensional coordinates corresponding to the point, suggesting that EL increases at the point where direction and size of velocity vectors change. Based on the time-flow curve and synchronized electrocardiogram, each cardiac cycle was divided into 5 phases (Fig. 1): isovolumic relaxation (IVR), early diastole (ED), late diastole (LD), isovolumic contraction (IVC), and ejection (EJE). The total diastole (TD) includes IVR, ED and LD; while the total systole (TS) includes IVC and EJE. The left ventricular EL during different phases were collected and averaged in three cardiac cycles. Additionally, the EL in this study indicated the average EL, its value was determined as the total EL divided by the area of the region of interest.

### Reproducibility

Approximately 15% of all of the participants were randomly selected to evaluate the intra-observer and inter-observer variabilities. The intra-observer variability was assessed by repeating the measurements on two different occasions. The inter-observer variability was assessed by performing new measurements by a second examiner independently. Then intra-observer and inter-observer variability were analyzed using Bland-Altman bias plots.

### Statistical analysis

All statistical data were analyzed using SPSS 21.0 (SPSS, Inc., Chicago, IL). Normality was evaluated by the Kolmogorov-Smirnov test. Continuous data were expressed as the mean ± Standard Deviation (SD) or median and inter-quartile ranges. Comparisons between two groups were evaluated using Student’s t-test (non-directional). Comparisons between multiple groups were analyzed by One-way ANOVAs followed Tukey’s post hoc test (LSD test where equal variances were assumed, and Dunnett’s C test where equal variances were not assumed) Additionally, the Spearman correlation test and Multiple linear regression analysis (stepwise method) were used to estimate the potential variables that were associated with the diastolic EL or systole EL of CKD patients. All analyses were two-tailed, and *P* <  0.05 was considered statistically significant.

## Results

### Baseline clinical characteristics and conventional echocardiography

Baseline clinical characteristics and conventional echocardiographic were shown in Table 1. The patients with stages 1–3 CKD had similar age, sex, heart rate, blood glucose, total cholesterol and low-density lipoprotein as the control participants. The systolic blood pressure (SBP, *P* <  0.001), diastolic blood pressure (DBP, *P* = 0.001), serum creatinine (*P* <  0.001), creatinine clearance rate (*P* <  0.001), Triglycerides (*P* <  0.001), high-density lipoprotein (*P* <  0.001), albumin (*P* <  0.001), hemoglobin (*P* = 0.010) and body mass index (*P* = 0.004) were increased in early CKD patients compared to that in control participants. The above indices between CKD subgroups were comparable, except for SBP and DBP. The stage 1–3 CKD patients with poorly controlled hypertension (P-HTN) had higher SBP (*P* <  0.001) and DBP (*P* = 0.003) than that in the stage 1–3 CKD patients without hypertension (N-THN). Moreover, the stage 1–3 CKD patients with poorly controlled hypertension had raised SBP (*P* <  0.001) and DBP (*P* <  0.001) compared to the stage 1–3 CKD patients with well-controlled hypertension (W-HTN). In present study, the poorly controlled hypertension was found in 41.67% (20 / 48) of early CKD patients. Patients with stages 1–3 CKD had similar LVEDd, LVEDV and LVEF as control participants. Compared with the controls, stage 1–3 CKD patients had enhanced LVPW (*P* <  0.001), this change was particularly significant in stage 1–3 CKD patients with poorly controlled hypertension (P-HTN vs. N-HTN, *P* = 0.003 and P-HTN vs. W-HTN, *P* = 0.039). Likewise, stage 1–3 CKD patients had higher LVMI than that in controls (*P* = 0.001), the highest LVMI was found in early CKD patients with poorly controlled hypertension among the CKD subgroups (P-HTN vs. N-HTN, *P* <  0.001 and P-HTN vs. W-HTN, *P* = 0.001). The data of LVPW and LVMI suggested heart configuration changed more obviously in stage 1–3 CKD patients with poorly controlled hypertension. Furthermore, A wave and E/e’ increased in patients with stages 1–3 CKD compared to control participants (all *P* < 0.001), but had no statistical significance among the CKD subgroups (all *P* > 0.05). We also found that there was no difference between CKD patients and control groups about E-wave, but the CKD patients with poorly controlled hypertension had increased E-wave compared the other two subgroups (all *P* < 0.001).

**Table 1:** Primary Clinical and Echocardiographic Characteristics of the Participants

Parameters	CKD Patients (stage 1–3)				Control (*n* = 41)
	All (*n* = 48)	N-HTN (*n* = 10)	W-HTN (*n* = 18)	P-HTN (*n* = 20)	
Age (years)	50.81 ± 12.41	48.10 ± 14.39	50.94 ± 13.07	52.05 ± 11.16	44.49 ± 11.53
Male (all)	28(48)	5 (10)	12 (18)	11 (20)	23 (41)
BMI (kg/m^2^)	25.57 ± 3.95*	24.67 ± 2.30	25.84 ± 4.04	26.27 ± 4.36	23.33 ± 2.92
HR (beats/min)	75.85 ± 11.03	77.50 ± 12.14	77.61 ± 9.91	73.45 ± 11.52	71.49 ± 9.52
SBP (mmHg)	138.19 ± 19.80*	125.40 ± 7.50	125.67 ± 2.18	155.85 ± 15.72^& #^	118.12 ± 11.03
DBP (mmHg)	83.96 ± 12.11*	79.30 ± 11.92	77.39 ± 6.16	92.20 ± 11.74^& #^	75.68 ± 11.50
BG (mmol/L)	5.01 ± 1.01	4.67 ± 0.52	4.87 ± 0.87	5.31 ± 1.23	4.78 ± 0.48
TC (mmol/L)	5.13 ± 1.42	5.55 ± 1.22	4.71 ± 1.65	5.31 ± 1.23	4.75 ± 0.70
TG (mmol/L)	1.94 ± 1.10*	1.75 ± 0.90	1.95 ± 1.28	2.03 ± 1.04	1.22 ± 0.38
HDL (mmol/L)	1.19 ± 0.36*	1.26 ± 0.47	1.07 ± 0.29	1.18 ± 0.30	1.48 ± 0.32
LDL (mmol/L)	3.10 ± 0.83	3.46 ± 0.90	2.87 ± 0.64	3.12 ± 0.91	2.97 ± 0.55
SCR (μmol/L)	267.02 ± 182.95*	201.42 ± 111.90	299.52 ± 245.93	270.57 ± 139.53	65.31 ± 10.88
CCR (ml/min)	41.20 ± 24.07*	44.76 ± 19.81	40.81 ± 24.15	39.78 ± 26.76	123.72 ± 27.99
ALB (g/l)	34.09 ± 4.79*	31.24 ± 6.03	35.03 ± 3.72	34.66 ± 4.66	45.18 ± 2.78
HB (g/l)	128.08 ± 17.15*	128.20 ± 18.93	127.72 ± 14.36	128.35 ± 19.30	140.22 ± 15.01
LVEDd (mm)	48.32 ± 5.30	47.20 ± 4.24	47.44 ± 4.71	49.68 ± 6.16	46.85 ± 3.79
IVS (mm)	10.60 ± 2.20	9.00 ± 1.83	10.67 ± 2.30	11.35 ± 1.93	8.26 ± 1.00
LVPW (mm)	9.33 ± 1.85*	8.20 ± 0.92	8.78 ± 1.31	10.40 ± 2.09^& #^	8.05 ± 0.90
LVEDV (ml)	111.10 ± 30.75	103.50 ± 20.68	106.39 ± 25.27	119.15 ± 38.02	103.07 ± 18.83
LVEF (%)	66.12 ± 6.16	66.09 ± 5.01	66.27 ± 4.95	66.05 ± 4.78	66.73 ± 4.34
LVMI (g/m^2^)	92.02 ± 5.46*	74.00 ± 15.40	82.28 ± 24.20	109.80 ± 43.37^& #^	72.05 ± 11.84
E (cm/s)	70.27 ± 17.04	63.40 ± 7.73	63.73 ± 15.99	79.60 ± 17.44^& #^	69.57 ± 12.24
A (cm/s)	77.47 ± 15.78*	69.90 ± 15.00	75.52 ± 17.76	83.02 ± 12.68	56.74 ± 15.19
E/e’	10.38 ± 3.33*	8.51 ± 1.32	10.34 ± 3.57	11.76 ± 3.04	6.07 ± 1.81

Date given as mean ± Standard Deviation or number (total)

*ALB* Albumin, *A* Mitral late filling wave peak velocity, *BG* Blood glucose, *BMI* Body mass index, *CCR* Creatinine clearance rate, *CKD* Chronic kidney disease, *DBP* Diastolic blood pressure, *E* Mitral early filling wave peak velocity, *e’* Early mitral annular peak velocity on septal side, *HDL* High-density lipoprotein, *HB* Hemoglobin, *HR* Heart rate, *LDL* Low-density lipoprotein, *LVEDd* Left ventricular end-diastolic diameter, *LVEDV* Left ventricular end-diastolic volume, *LVEF* Left ventricular ejection fraction, *LVMI* Left ventricular mass index, *LVPW* Left ventricular posterior wall, *IVS* Interventricular septal thickness, *N-HTN* CKD patients (stage1–3) who had no hypertension, *P-HTN* CKD patients (stage1–3) who had poorly controlled hypertension, *SBP* Systolic blood pressure, *SCR* Serum creatinine, *TC* Total cholesterol, *TG* Triglycerides, *W-HTN* CKD patients (stage1–3) who had well-controlled hypertension

^*^P < 0.05 vs. control; ^&^*P* < 0.05 vs. N-HTN; ^#^*P* < 0.05 vs. W-HTN)

### Quantitative analysis of left ventricular energy loss

**Fig. 2:**
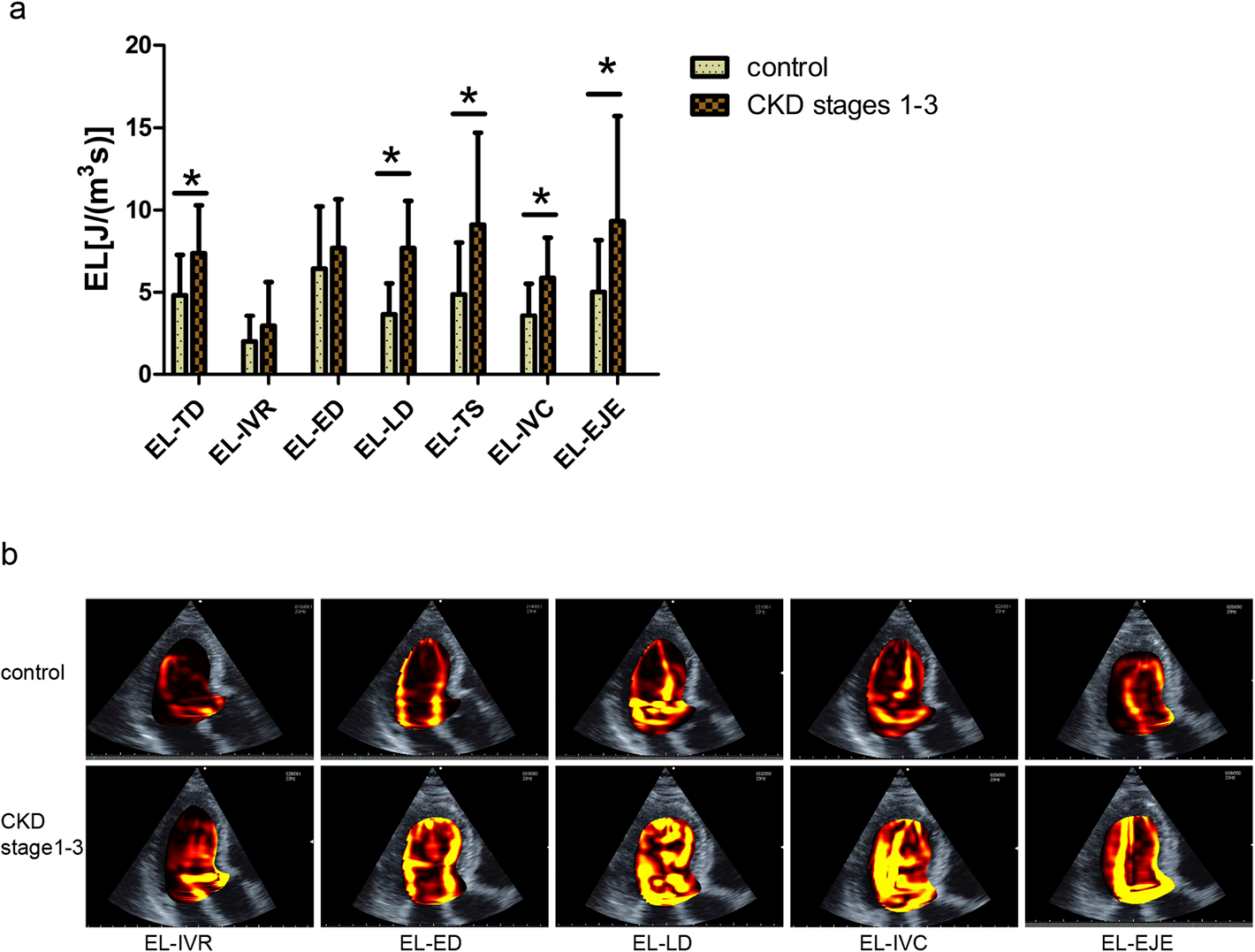
Left ventricular energy loss analysis between patients with stages 1–3 CKD and controls. **a** Compared to control participants, patients with stages 1–3 CKD had high left ventricular EL-TD, EL-LD, EL-TS, EL-IVC and EL-EJE. **b** Representative images of EL-IVR, EL-ED, EL-LD, EL-IVC and EL-EJE. The higher energy loss values are demonstrated by bright yellow. The images were respectively collected from a control participant or a patient. (*P < 0.05)

The date of left ventricular EL were shown in Table 2. Compared to control participants, patients with stages 1–3 CKD had increased EL during total diastole phase (EL-TD) (*P* < 0.001) and raised EL during total systole phase (EL-TS) (*P* < 0.001). Those data suggested patients with stages 1–3 CKD had more intraventricular energy dissipation than healthy participants. Similarly, EL during late diastole phase (EL-LD), EL during isovolumic contraction phase (EL-IVC) and EL during ejection phase (EL-EJE) were found increased in patients with stages 1–3 CKD compared to control participants (all *P* < 0.001). However EL during isovolumic relaxation phase (EL-IVR) and EL during the early diastole phase (EL-ED) had no significant difference between patients with stages 1–3 CKD and control participants (*P* > 0.05). The analysis of left ventricular EL between CKD and control participants was illustrated in Fig. 2a. Moreover, representative images of EL-IVR, EL-ED, EL-LD, EL-IVC and EL-EJE from a control or a CKD participant were shown in Fig. 2b. More EL was indicated by bright yellow in images.

**Table 2:** Left ventricular EL during different phase of cardiac cycle

Parameters [J/(m^3^s)]	CKD patients (stage1–3)				Control (*n* = 41)
	All (*n* = 48)	N-HTN (*n* = 10)	W-HTN (*n* = 18)	P-HTN (*n* = 20)	
EL-IVR	2.92 ± 2.70	1.97 ± 0.83	2.74 ± 2.22	3.55 ± 3.51	1.97 ± 1.58
EL-ED	7.65 ± 2.97	5.17 ± 1.66	5.45 ± 3.84	9.84 ± 4.59	6.45 ± 3.78
EL-LD	7.65 ± 2.87	6.79 ± 1.90	6.85 ± 2.63	9.29 ± 2.68	3.65 ± 1.89
EL-TD	7.38 ± 2.91	6.47 ± 1.33	6.56 ± 3.06	8.58 ± 3.03	4.83 ± 2.45
EL-IVC	5.87 ± 2.45	4.27 ± 2.48	5.53 ± 2.63	7.00 ± 1.71	3.58 ± 1.94
EL-EJE	9.33 ± 6.36	6.09 ± 2.38	7.18 ± 2.82	12.89 ± 8.18	5.02 ± 3.15
EL-TS	9.10 ± 5.61	5.65 ± 2.10	7.02 ± 4.54	12.70 ± 5.78	4.88 ± 3.15

Date given as mean ± Standard Deviation

*EL* Energy loss, *EL-ED* Energy loss during early diastole, *EL-LD* Energy loss during late diastole, *EL-EJE* Energy loss during ejection phase, *EL-IVC* Energy loss during isovolumic contraction phase, *EL-IVR* Energy loss during isovolumic relaxation phase, *EL-TD* Energy loss during total diastole, *EL-TS* Energy loss during total systole

**Fig. 3:**
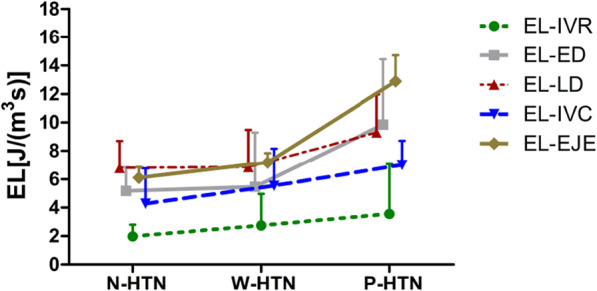
The trend of energy loss in CKD subgroups. EL-IVR, EL-ED, EL-LD, EL-IVC and EL-EJE showed increased trend in in CKD subgroups

**Fig. 4:**
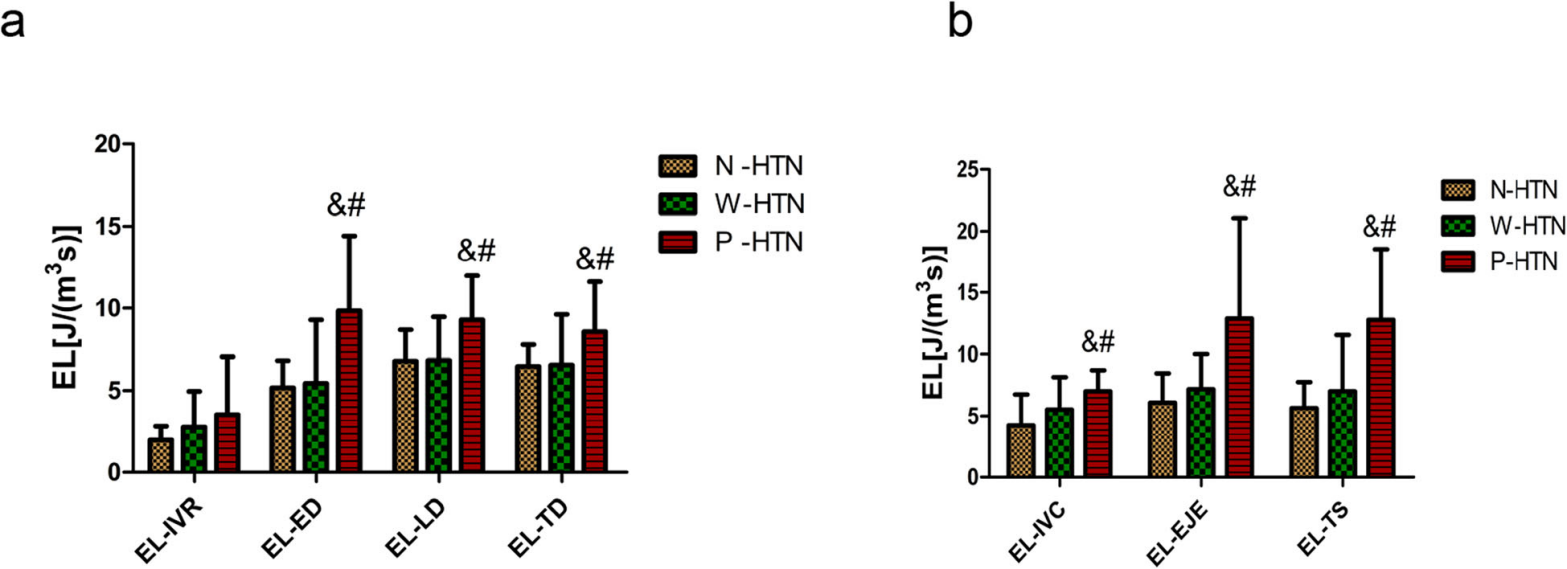
Left ventricular energy loss analysis between CKD subgroups. **a** EL-IVR, EL-ED, EL-LD and EL-TD in each CKD subgroup were shown in histogram. EL-ED, EL-LD and EL-TD increased in P-HTN. **b** EL-IVC, EL-EJE and EL-TS in each CKD subgroup were shown in histogram. Compared to other two groups, P-HTN had the high left ventricular EL-TS, EL-IVC and EL-EJE. (^&^*P* < 0.05 vs. N-HTN; ^#^*P* < 0.05 vs. W-HTN)

Interestingly, EL-IVR, EL-ED, EL-LD, EL-IVC and EL-EJE all presented increased trend in CKD subgroups (shown in Fig. 3), suggesting a possible influence of hypertension on left ventricular EL. To explore whether the hypertension have further effect on the blood flow efficiency in patients with stages 1–3 CKD, left ventricular EL were analyzed in CKD subgroups (shown in Fig. 4). Notably, the EL-TD enhanced more obviously in patients with poorly controlled hypertension (P-HTN vs. N-HTN, *P* = 0.041; P-HTN vs. W-HTN, *P* = 0.020). Similarly, EL-LD raised more significantly in the patients with poorly controlled hypertension (P-HTN vs. N-HTN, *P* = 0.005; P-HTN vs. W-HTN, *P* = 0.001) and the increased EL-ED was also found in patients with poorly controlled hypertension (P-HTN vs. N-HTN, *P* = 0.016; P-HTN vs. W-HTN, *P* = 0.018). However, EL-IVR had no significant difference between the subgroups (shown in Fig. 4a). Except for the diastolic EL, we also analyzed the systolic EL between the three subgroups of patients with stages 1–3 CKD (shown in Fig. 4b). The obviously increased EL-TS were found in patients with poorly controlled hypertension (P-HTN vs. N-HTN, *P* < 0.001; P-HTN vs. W-HTN, *P* < 0.001). Next, we explored EL-IVC had a similar statistical result to EL-TS between the three subgroups (P-HTN vs. N-HTN, *P* = 0.001; P-HTN vs. W-HTN, *P* = 0.036), and the higher level of EL-EJE was also detected in patients with poorly controlled hypertension (P-HTN vs. control, *P* = 0.003; P-HTN vs. N-HTN, *P* = 0.013; P-HTN vs. W-HTN, *P* = 0.042). Together, those data suggested patients with stages 1–3 CKD had increased EL during diastole and systole, this hemodynamic abnormality was more significant in CKD patients with poorly controlled hypertension.

### Correlation and stepwise multiple regression analysis

The results of correlation analysis were shown in Table 3. In the patients with early CKD, the diastolic EL was significantly associated with the E/e’ ratio (*r* = 0.744, *P* < 0.001), E-wave (*r* = 0.732, *P* < 0.001), A-wave (*r* = 0.510, *P* < 0.001), LVMI (*r* = 0.478, *P* = 0.001) and LVPW (*r* = 0.428, *P* = 0.002), whereas systolic EL was significantly associated with LVMI (*r* = 0.764, *P* < 0.001) and SBP (*r* = 0.663, *P* < 0.001).The results of stepwise multiple regression analysis were shown in Table 4. In the patients with early CKD, E/e’, E-wave and LVMI were independent predictors of the EL-TD, whereas SBP and LVMI were independent predictors of the EL-TS. Additionally, the regression equation obtained was as follows: EL-TD = − 3.356 + 0.306E/e’ + 0.088E + 0.018LVMI (adjusted R^2^ 0.693, *P* < 0.001), EL-TS = − 10.420 + 0.090LVMI + 0.081SBP (adjusted R^2^ 0.614, *P* < 0.001).

**Table 3:** Correlation between left ventricular energy loss and other variables in stage1–3 CKD patients

Variables	EL-TD		EL-TS	
	Correlation coefficient (r)	*P*	Correlation coefficient (r)	*P*
E/e’	0.741	< 0.001	0.383	0.007
A	0.504	< 0.001	0.361	0.012
E	0.732	< 0.001	0.097	0.514
LVMI	0.604	< 0.001	0.561	< 0.001
SBP	0.321	0.026	0.583	< 0.001

**Table 4:** Multivariate regression equation for predicting left ventricular energy loss in patients with CKD stages 1–3

Variables	EL-TD			EL-TS		
	Coefficient	Standard error	*P*	Coefficient	Standard error	*P*
(Intercept)	−3.356	1.117	0.004	−10.420	3.836	0.009
E/e’	0.306	0.100	0.004			
E	0.088	0.019	< 0.001			
LVMI	0.018	0.007	0.019	0.090	0.019	< 0.001
SBP				0.081	0.034	0.021
	Residual SE 1.613			Residual SE 3.664		
	Adjusted R^2^ 0.693			Adjusted R^2^ 0.614		
	*P* < 0.001			*P* < 0.001		

### Intra-observer and inter-observer variability

**Fig. 5:**
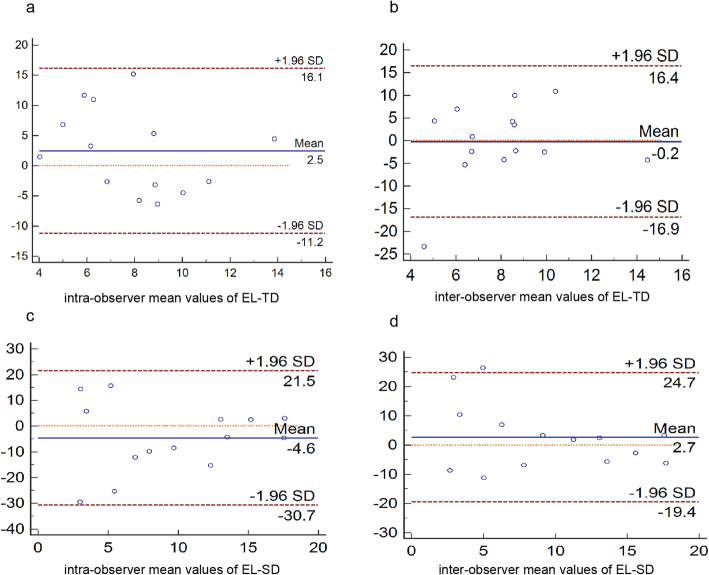
Bland-Altman plots of intra-observer and inter-observer variability. **a** intra-observer variability of energy loss during total diastole. **b** inter-observer variability of energy loss during total diastole. **c** intra-observer variability of energy loss during total systole. **d** inter-observer variability of energy loss during total systole. Numerical unit of EL: [J/(m^3^s)]

The Bland-Altman analysis illustrated that measurements of left ventricular EL-TD and EL-TS exhibit good reproducibility (Fig. 5)

## Discussion

Intracardiac blood flow status has not been investigated in stage 1–3 CKD. In current study, the left ventricular EL in CKD stages 1–3 with primary glomerulonephritis were quantitatively analyzed using VFM technology. We found that patients with stages 1–3 CKD had increased left ventricular EL during diastole and systole, this hemodynamic abnormality was more significant in CKD patients with poorly controlled hypertension. Using correlation and multivariate regression analysis, we indicated blood pressure might play some role in the increased EL.

VFM, as a novel echocardiographic technology, has been shown to be capable to quantitatively evaluate EL derived from the velocity vector field of the blood flow in ventricle [12]. EL value is supposed to demonstrate the impact of the viscous dissipation on cardiac change adapted to a physical condition [20]. There was an increased diastolic EL in patients with stages 1–3 CKD, suggesting that this population had more intraventricular energy dissipation and less flow efficiency than the healthy participants. In present study, when the E-wave velocity or A-wave velocity was increased, the EL during diastole tended to be high. As the EL value is calculated from an equation, EL increases at the point where direction and size of velocity vectors change [25]. If E-wave velocity or A-wave velocity increases, the rapid influx of blood flow makes a strong collision on the blood flow remained in the heart cavity, which results in changes of the velocity and direction of the blood flow, and more energy dissipation. Furthermore, the increase of myocardial collagen content and myocardial stiffness in early-stage CKD cause a reduction of active myocardial relaxation, then lead to a deterioration of diastolic function and a raise of filling pressure [26]. E/e’ ratio was reported to have quality to predict left ventricular filling pressure [27] and be a sensitive tool to detect diastolic function [28]. In our study, the patients with stages 1–3 CKD had increased E/e’ ratio, which was consistent with the previous data [29]. Moreover, the E/e’ ratio was shown as a predictor of diastolic EL in our data, indicating that diastolic dysfunction and increased filling pressure might play some role in the energy dissipation. From another point of view, when diastolic dysfunction happens, blood flow loses more energy during diastolic to achieve sufficient left ventricular filling, ensuring sufficient cardiac output. Therefore, we suggested EL might be a novel echocardiographic parameter for evaluating diastolic dysfunction in patients with stages 1–3 CKD from the perspective of hemodynamics. Which was consistent with the opinion in a previous study about EL of diabetic patients [15]. Additionally, the pathological cardiac configurations, including abnormal heart size and ventricular wall thickness, transform blood flow status from the uniform laminar pattern into a chaotic one, causing increased EL. In present study, the LVMI had close relation with the diastolic EL in early CKD patients and been indicated as an independent predictor of the diastolic EL, suggesting a relation of hemodynamic abnormalities and pathological cardiac configuration in this population. Furthermore, we found that EL during late diastole showed similar results as total diastole. But EL during early diastole had no significant difference between the patients with early CKD and control participants, which may be attribute to the fluctuation of EL during early diastole with different degrees of diastolic dysfunction in patients [30].

Since the cardiac cycle is continuous, abnormality in blood flow can last from diastole to systole. It had been confirmed by VFM in patients with a reduced LVEF [31], in whom the abnormal intraventricular vortex last from diastole to systole. Physiologic vortex starts at the ventricle side the mitral valve in diastolic phase, then passes through the isovolumetric contraction phase and finally disappears in the ejection phase [32]. The vortex turns the blood flow from the left ventricular inflow tract to the outflow tract, effectively transferring energy and avoiding excessive dissipation of energy. On the contrary, the pathological vortex displays a scattered distribution and a long duration [33], dissipates more energy, resulting in an increased EL. In addition to the elevated diastolic El, our data demonstrated that patients with early CKD had increased systolic EL during total systole, isovolumic contraction and ejection phase, suggesting a coexistence of impaired blood flow efficiency in diastole and systole, which was consistent with a previous research [20]. We speculated that the increased systolic EL may be a continuation of the hemodynamic abnormality in diastole. Additionally, Frank-Starling mechanism is a coupling mechanism accepted widely between afterload and cardiac contraction. When afterload increases, the contractility of left ventricle increases to ensure a sufficient ejection volume [34]. The increased contraction of left ventricle intensifies the interaction between blood flows, as well as the blood flow and ventricular wall [35], causing more energy dissipation. Moreover, SBP, a clinical indicator representing cardiac afterload, was shown as the predictor of systolic EL in patients with stages 1–3 CKD in present study. We considered the mechanism of the increased systolic EL in patients with stages 1–3 CKD may be a compensatory of the Left ventricular blood flow in response to the elevated afterload. Importantly, LVMI was closely associated with systolic EL, and shown as a predictor of the systolic EL in the patients with stages 1–3 CKD, suggesting pathological cardiac configuration contributes to the abnormality of left ventricular blood flow during systole [33, 36]. Overall, the increased systolic EL coexisted with the increased diastolic EL in patients with stages 1–3 CKD, the mechanism of systolic EL may be a continuation of the hemodynamic abnormality in diastole, or a hemodynamic compensatory mechanism in response to afterload, or related to the pathological cardiac configuration due to CKD.

Patients with stages 1–3 CKD generally have no serious complications and frequently experience hypertension, edema, proteinuria and anemia, leading to hypoproteinemia and increased BMI. Using correlation and stepwise multiple regression analysis, we found only the SBP was the predictor of systolic EL in patients with stages 1–3 CKD, suggesting that the clinical presentation had little bearing on the result of assessment of left ventricular EL. Hypertension, as a main cardiovascular risk factor, is a main clinical symptom in patients with stages 1–3 CKD. However, it does not get enough attention in real-world. In present study, 41.67% of patients had poorly controlled hypertension, suggesting a poor condition of blood pressure management in CKD during the early stages. Unexpectedly, our data confirmed stage 1–3 CKD patients with poorly controlled hypertension had higher left ventricular EL compared to those patients with well-controlled hypertension or with no hypertension, indicating hypertension was a crucial contributor for intracardiac blood flow abnormality in early-stage CKD. Hypertension can increase cardiac afterload and damage cardiac configuration in the background of CKD, then further deteriorate hemodynamic abnormalities. Blood pressure management, is considered as the main target of CKD treatment. Our date revealed the importance of hypertension control from the perspective of hemodynamics in early-stage CKD when the best treatment time window exists. Considering the harmfulness of cardiovascular complications and the high prevalence of poorly controlled hypertension, Patients with CKD stage 1–3 are supposed to pay more attention to their blood pressure level.

The present research has several limitations. Although CKD related to diabetes has become more prevalent than CKD related to glomerulonephritis in China, the recruited CKD participants in current study were only patients with primary glomerulonephritis. This relatively single patient population may hinder the universalization of our findings in all CKD patients. Additionally, this work was a single-center cross-sectional study with a relatively small sample size, which might contribute to the result (without statistical difference) of left ventricular EL during isovolumic diastole. The changes in left ventricular EL values in patients with stages 1–3 CKD should be validated by further multi-center, large-scale longitudinal studies. Furthermore, Although the recruited CKD participants all accepted professional treatment according to CKD clinical guidelines, we did not evaluate the influence of the treatment protocols on the results in this cross-sectional study without therapeutic intervention.

## Conclusion

In conclusion, left ventricular EL was a practical echocardiographic parameter to evaluate the impaired ventricular blood flow efficiency exists in patients with early-stage CKD. Poorly controlled hypertension was a crucial contributor for intracardiac blood flow abnormality. We expected that this study provides hemodynamic evidence to reduce the disproportionately risk of cardiovascular disease and slow, or halt the progression of CKD during the early stages.

## Data Availability

The datasets generated and analyzed in this study are available from the corresponding author on request.

## Abbreviations

A: Mitral late filling wave peak velocity
CKD: Chronic kidney disease
DBP: Diastolic blood pressure
E: Mitral early filling wave peak velocity
e*’*: Early mitral annular peak velocity on septal side
EL: Energy loss
EL-ED: Energy loss during early diastole
EL-LD: Energy loss during late diastole
EL-EJE: Energy loss during ejection phase
EL-IVC: Energy loss during isovolumic contraction phase
EL-IVR: Energy loss during isovolumic relaxation phase
EL-TD: Energy loss during total diastole
EL-TS: Energy loss during total systole
LVEDd: Left ventricular end-diastolic diameter
LVEDV: Left ventricular end-diastolic volume
LVEF: Left ventricular ejection fraction
LVMI: Left ventricular mass index
LVPW: Left ventricular posterior wall
IVS: Interventricular septal thickness
SBP: Systolic blood pressure
